# Supplementary material for: Materializing efficient methanol oxidation via electron delocalization in nickel hydroxide nanoribbon
Source: Nat Commun. 2020 Sep 16;11:4647. doi: 10.1038/s41467-020-18459-9 (PMC7495422; doi:10.1038/s41467-020-18459-9)
Supplement: Supplementary file 1 — Supplementary Information [file 41467_2020_18459_MOESM1_ESM.pdf]

Supplementary Information

# Materializing efficient methanol oxidation via electron delocalization in nickel hydroxide nanoribbon

Wang *et al*.

## Supplementary Figures

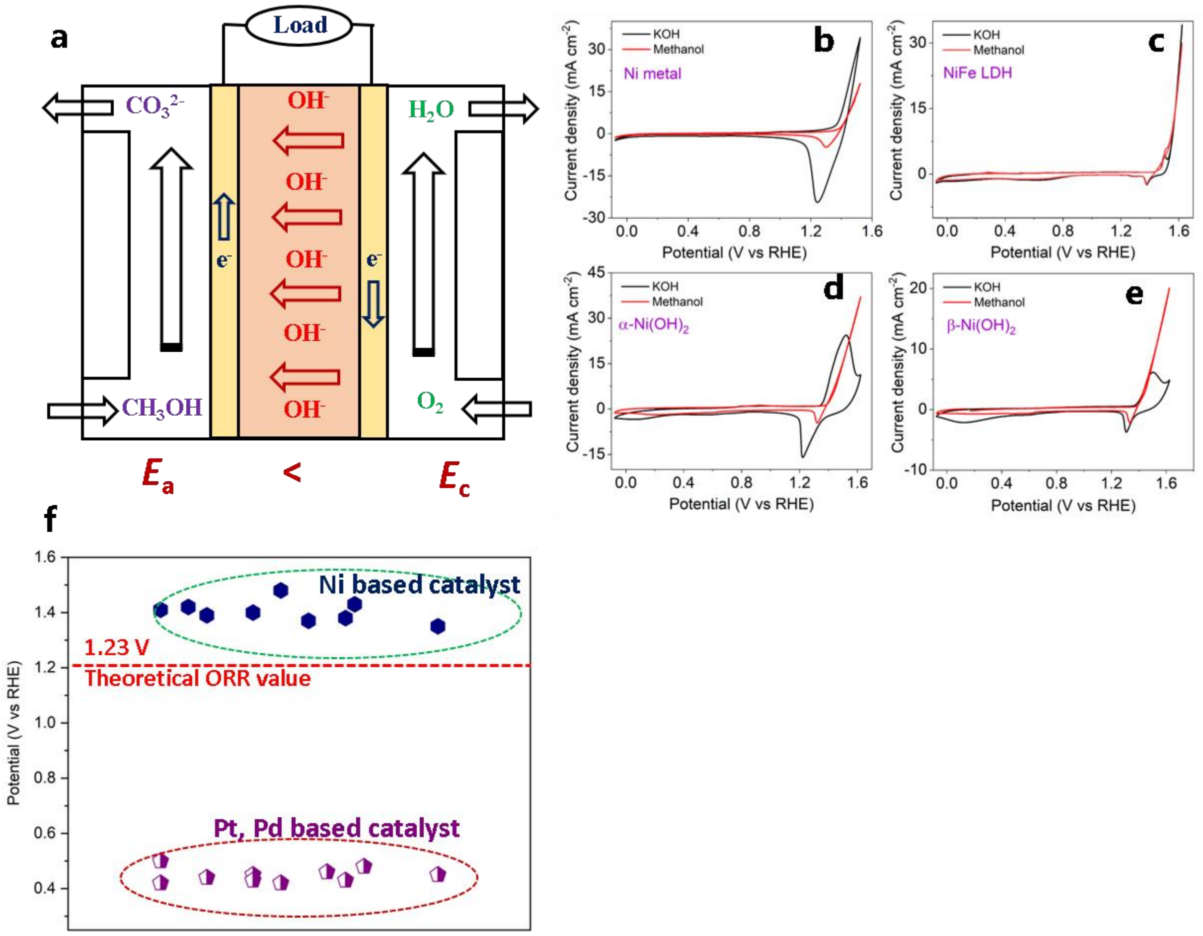


**Supplementary Figure 1. The MOR and DMFCs measurement of traditional Ni-based catalysts.** (a) the schematic of the DMFCs; (b) - (e) the MOR measurement of α/β-$Ni(OH)_2$, Ni metal, NiFe LDH catalysts; (f) the reported MOR potential of traditional Ni-based catalysts. In this work, the MOR activities of Ni metal, NiFe LDH and α/β-$Ni(OH)_2$ catalysts are evaluated by conducting CV scans from -0.1 to 1.6 V in 1M KOH + 1M $CH_3OH$ electrolyte solution using a three-electrode system (Supplementary Figure 1b to e). The results indicate that, the MOR potential for these catalysts are larger than 1.35 V vs RHE, which agrees well with the reported MOR potential values (Supplementary Figure 1f) (*1*-*3*).

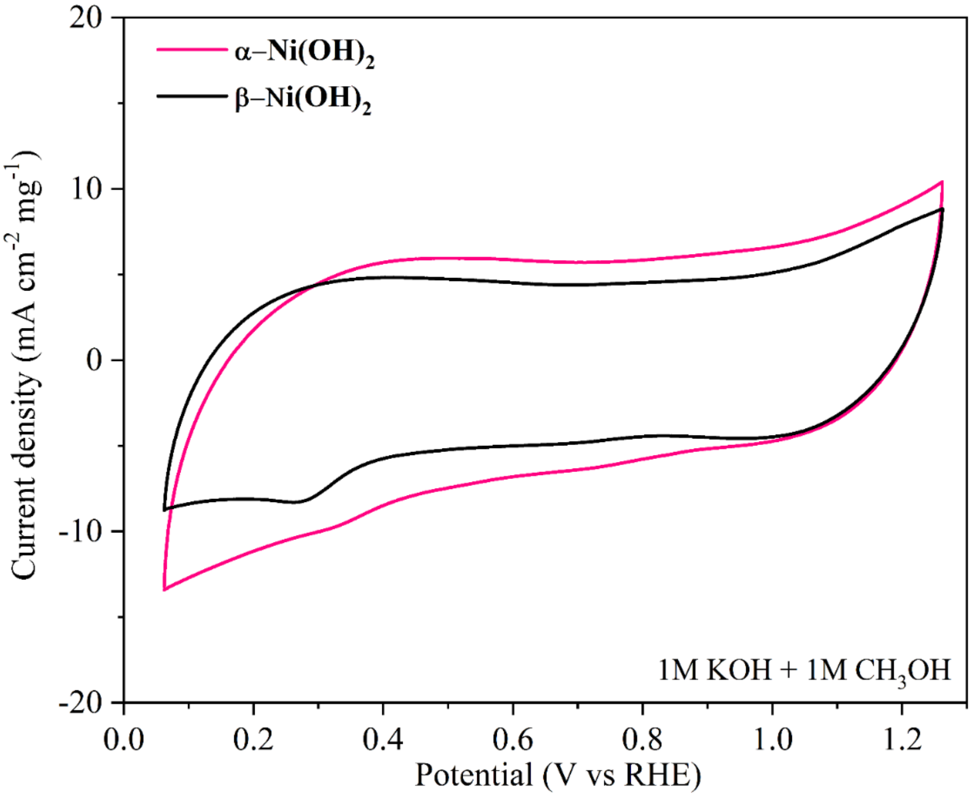


**Supplementary Figure 2. The MOR activity of α-$Ni(OH)_2$ and β-$Ni(OH)_2$ in the potential range from 0 V to 1.23V.** There is no MOR activity of these two control samples, suggesting that the excellent MOR activity of NR-$Ni(OH)_2$ arises from its unique nanoribbon structure.

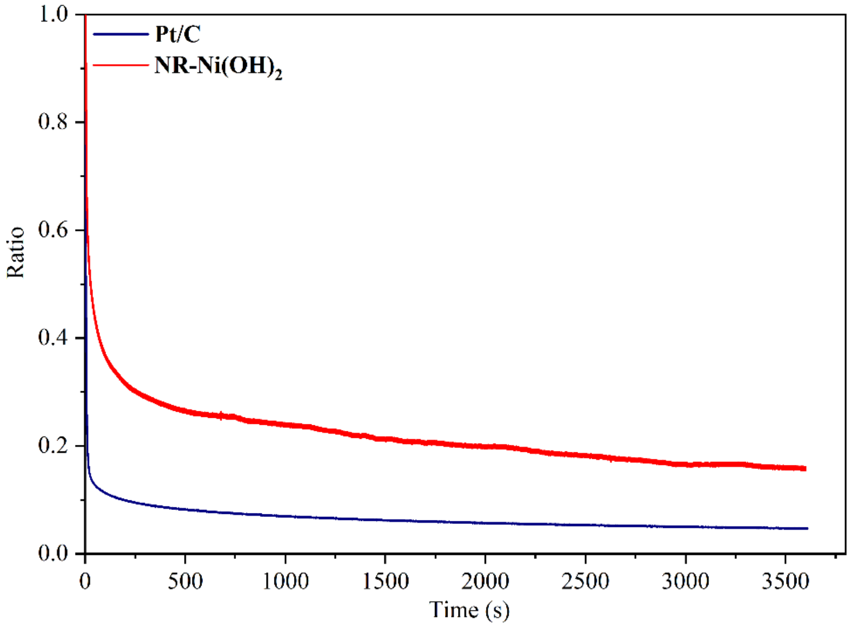


**Supplementary Figure 3. The operation durability test of NR-$Ni(OH)_2$ and Pt/C.** In this measurement, the potential values used were the forward potential peak (NR-$Ni(OH)_2$ 0.89V; Pt/C 0.79V). Over 3600s test, NR-$Ni(OH)_2$ exhibits 84% loss in activity, while Pt/C has 96% activity loss, suggesting that NR-$Ni(OH)_2$ has better stability than Pt/C.

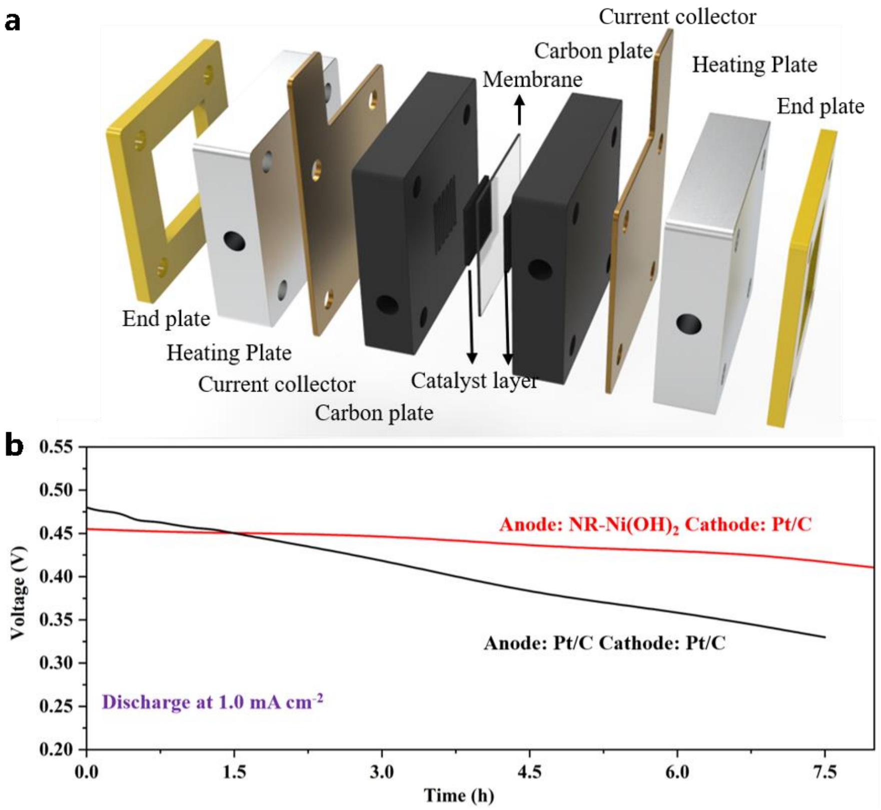


**Supplementary Figure 4. DMFC assemblies and Performance.** (a) the schematic of DMFCs devices; (b) Transient discharge voltage of DMFCs (black: Pt/C for both anode and cathode; red: NR-$Ni(OH)_2$ for anode and Pt/C for cathode). The device was operated at 80 ºC, with 1M methanol concentration and an $O_2$ flow rate of 190 cc/min.

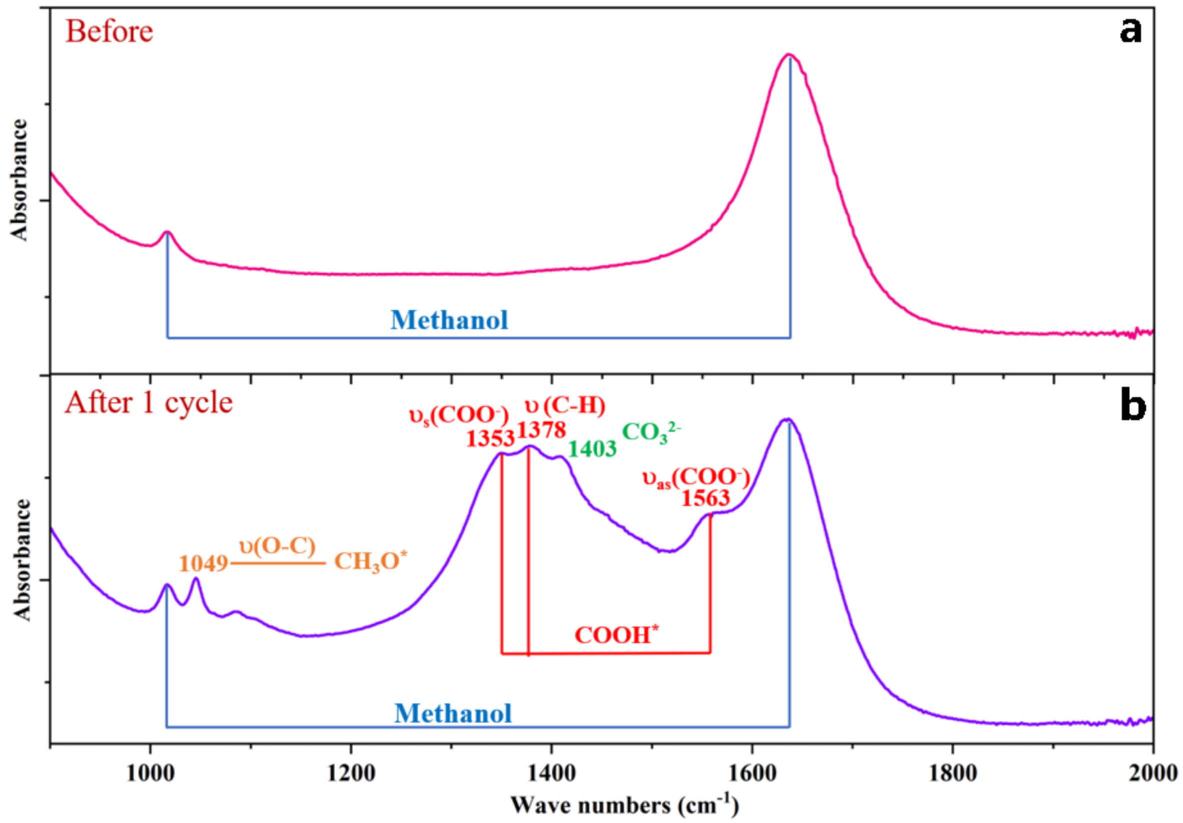


**Supplementary Figure 5. FTIR spectra of NR-Ni(OH)2/solution before (a) and after (b) electrochemical measurement.** The bands at 1049 $cm^{-1}$ are attributed to ν(O-C) stretching vibration of methoxy species ($CH_3O^*$); the bands at 1563 and 1353 $cm^{-1}$ correspond to asymmetric and symmetric $CO_2^-$ stretching of $COOH^*$, respectively; the 1378 $cm^{-1}$ peak corresponds to the C-H vibration in $COOH^*$; the 1403 $cm^{-1}$ peak represents $CO_3^{2-}$ species.

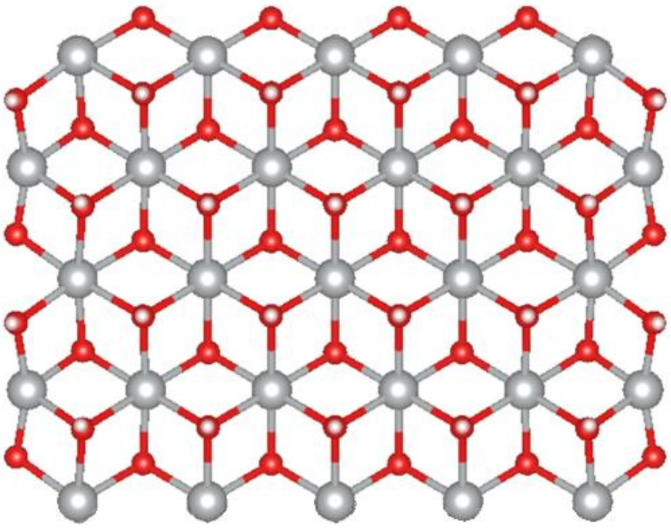

**Supplementary Figure 6. The atomic model of NR-Ni(OH)$_2$.** Here, we used a 2x1 supercell extended along the length direction to simulate adsorption of the intermediates. The gray, red and light blue balls represent Ni, O and H atoms, respectively. The width of NR is ~17.3 Å and with a vacuum thickness of 12 Å. The Ni atoms at the edge are the active sites, with unsaturated coordination of 4 $OH^-$.

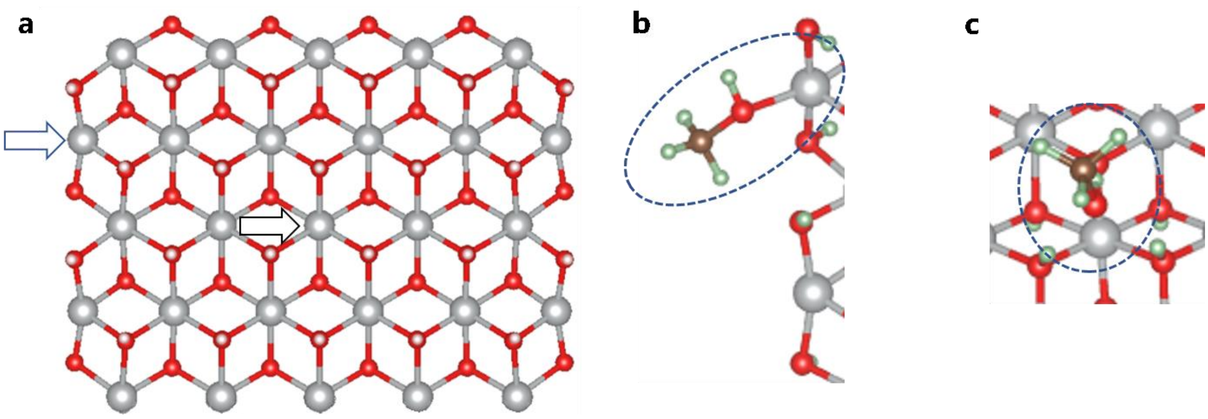


**Supplementary Figure 7. Atomic models of the NR-Ni(OH)$_2$ and the methanol molecule adsorption.** Supplementary Figure 7a shows two possible adsorption sites. Supplementary Figure 7b and c show the methanol molecule adsorption with four-fold Ni at NR edge site (Supplementary Figure 7b) and with six-fold Ni at the NR interior (Supplementary Figure 7c), respectively. Based on the symmetry of NR models as shown in Supplementary Figure 7a, there are two possible adsorption sites for methanol molecule adsorption. One is with the four-fold Ni at the NR edge site indicated by the blue arrow, and the other is the six-fold Ni at the interior indicated by the black arrow. We performed DFT calculations to investigate the adsorption energy using $E_{ads} = E_{NR+M} - (E_{NR} + E_M)$. Here, $E_{NR+M}$ , $E_{NR}$ and $E_M$ are the total energies of the NR with the adsorbed methanol molecule, without the adsorbed methanol molecule and an isolated methanol molecule, respectively. The calculated adsorption energies are -1.39 eV at the edge site and -0.32 eV at the internal site, respectively showing that the methanol molecule prefers to adsorb with Ni atoms at the edge site. Not surprisingly, Ni atoms located at the interior of the NR are fully saturated by six OH, while Ni atoms located at NR edges bond with four OH, leaving two dangling bonds, which are the edge sites active for methanol adsorption.

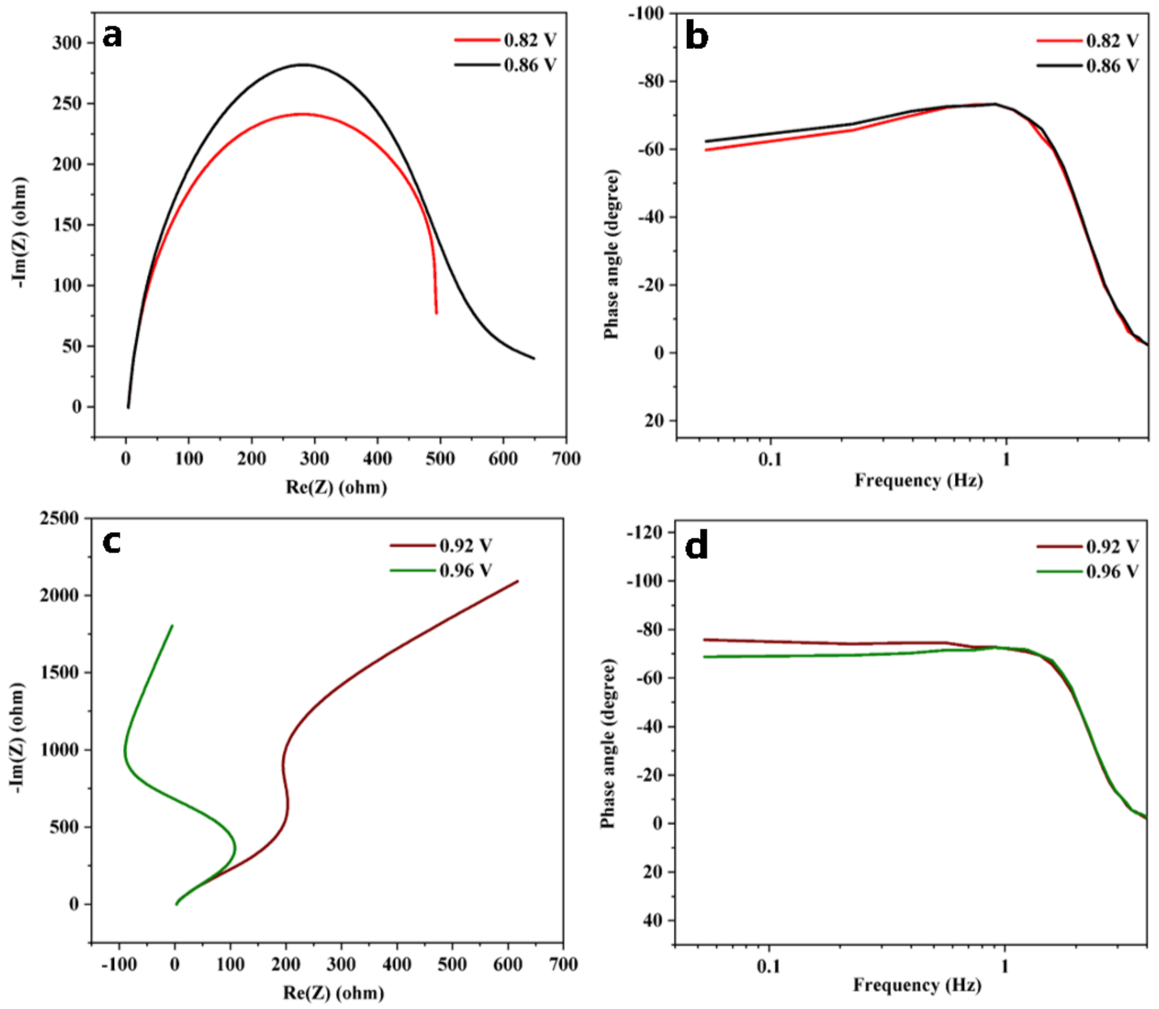


**Supplementary Figure 8. Nyquist and phase-frequency plots of NR-Ni(OH)₂.** (a) and (c): Nyquist plots measured at 0.82 V, 0.86 V, 0.92 V, and 0.96 V, respectively; (b) and (d) Bode plots measured at 0.82 V, 0.86 V, 0.92 V, and 0.96 V, respectively. When the potential was increased to 0.92 V, the impedance starts to appear in the second quadrant in the Nyquist plot and a new hump was observed in the frequency ranges from 0.1 to 1 Hz in the Bode plots (b, d). These behaviors are usually attributed to the change in the rate-determining step with applied potential. Similar cases could be found in the methanol oxidation using Pt/Ru electrode and glucose oxidation in the metal-modified glassy carbon (*4*).

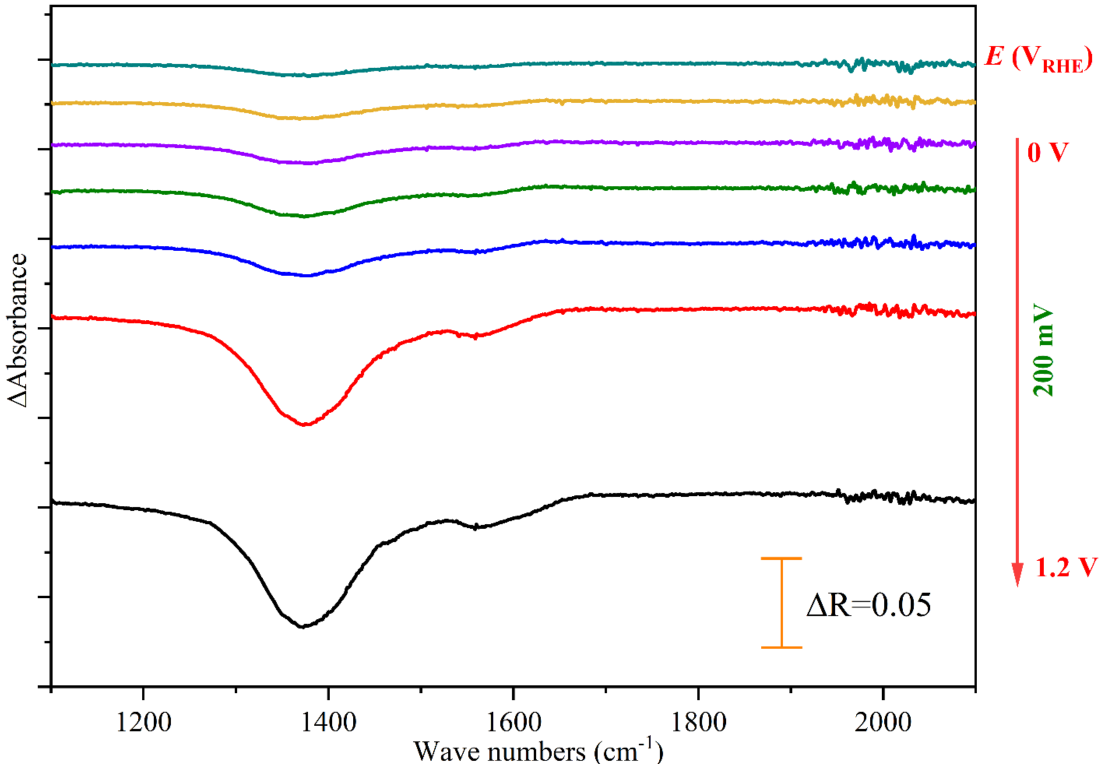


**Supplementary Figure 9.** *In-situ* FTIR of electrode surface with an applied voltage from 0 to 1.2V. There is no detectable CO attached on the electrode surface, confirming that there is negligible CO poisoning for NR-$Ni(OH)_2$.

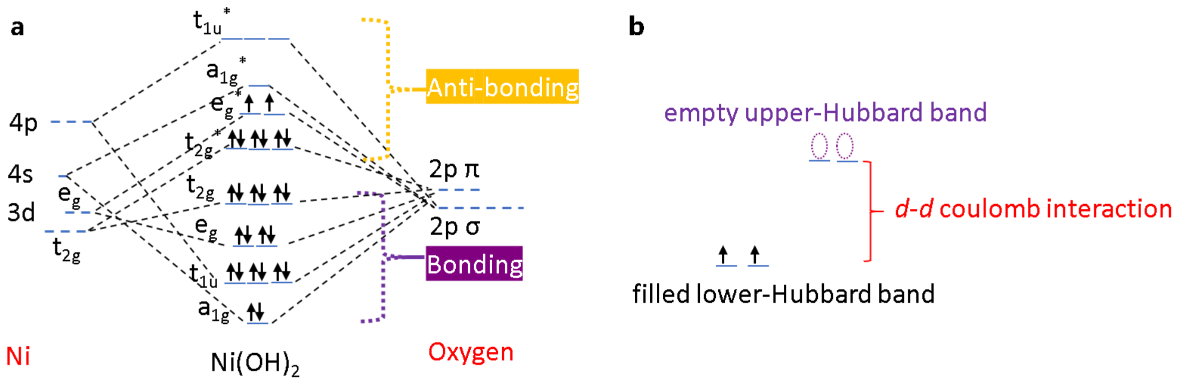


**Supplementary Figure 10.** (a) the schematic of traditional $Ni(OH)_2$ electronic orbitals; (b) the schematic of upper-Hubbard band (UHB) and lower-Hubbard band (LHB). In $Ni(OH)_2$ structure, the Ni-O bond is formed through the hybridization of O 2p orbitals and Ni 3p, 4s and 4d orbitals. The detailed molecular orbital diagram of $Ni(OH)_2$ is shown in Supplementary Figure 10a. It includes (M-O) bonding bands ($a_{1g}/t_{1u}/e_g/t_{2g}$) exhibiting oxygen character, and $(M\text{-}O)^*$ antibonding bands ($a_{1g}^*/t_{1u}^*/e_g^*/t_{2g}^*$) showing metal character. Among these bonding bonds, the (M-O) bonding bands and $t_{2g}^*$ orbital in $(M\text{-}O)^*$ antibonding bands are fully occupied by electrons, while the $e_g^*$ orbital in $(M\text{-}O)^*$ antibonding bands is partially occupied by electrons, with two spin up electrons on it. Due to the strong *d-d* coulomb interaction, this $e_g^*$ orbital splits in to empty upper-Hubbard band (UHB) and filled lower-Hubbard band (LHB). In this work, we ignore $t_{2g}^*$ orbital and just discuss $e_g^*$ orbitals.

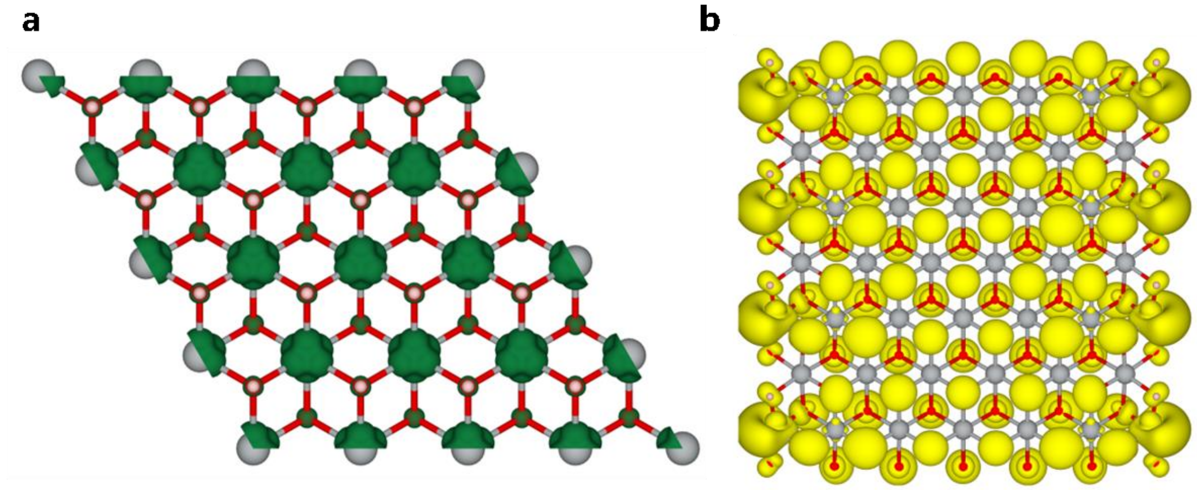


**Supplementary Figure 11.** (a) (b) the isosurface charge density of β-$Ni(OH)_2$ and NR-$Ni(OH)_2$ UHB, respectively. From this figure, we could clearly see the shapes of Ni and O edge atoms deformed and became larger in NR-$Ni(OH)_2$. This means, the UHB delocalization in NR-$Ni(OH)_2$ results from the four-coordinated Ni atoms.

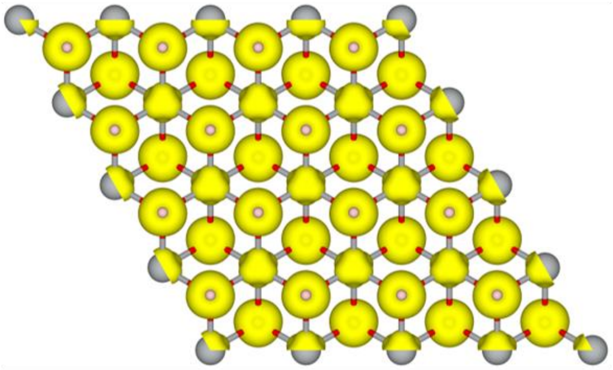

**Supplementary Figure 12.** The isosurface charge density of β-$Ni(OH)_2$ LHB. It indicates, there was no obvious shape deformation for different O and Ni atoms.

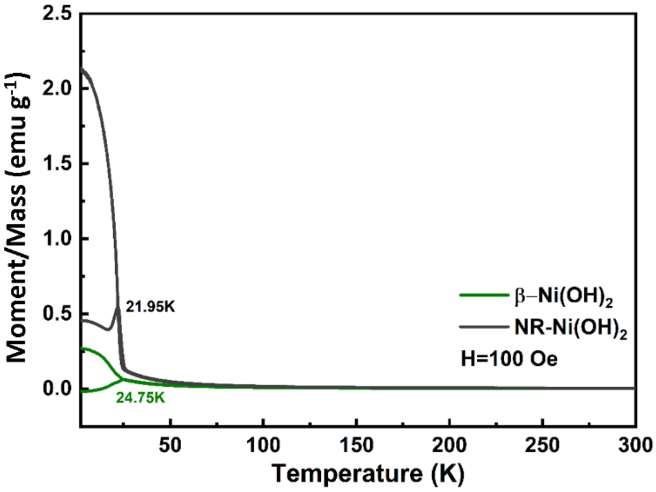


**Supplementary Figure 13.** ZFC (zero field cooled) and FC (field cooled) magnetizations for NR-$Ni(OH)_2$ and β-$Ni(OH)_2$ as function of temperature with applied magnetic field H= 100 Oe. It indicates, the Neel temperature ($T_N$) for NR-$Ni(OH)_2$ is 21.95 K, while β-$Ni(OH)_2$ is 24.75 K.

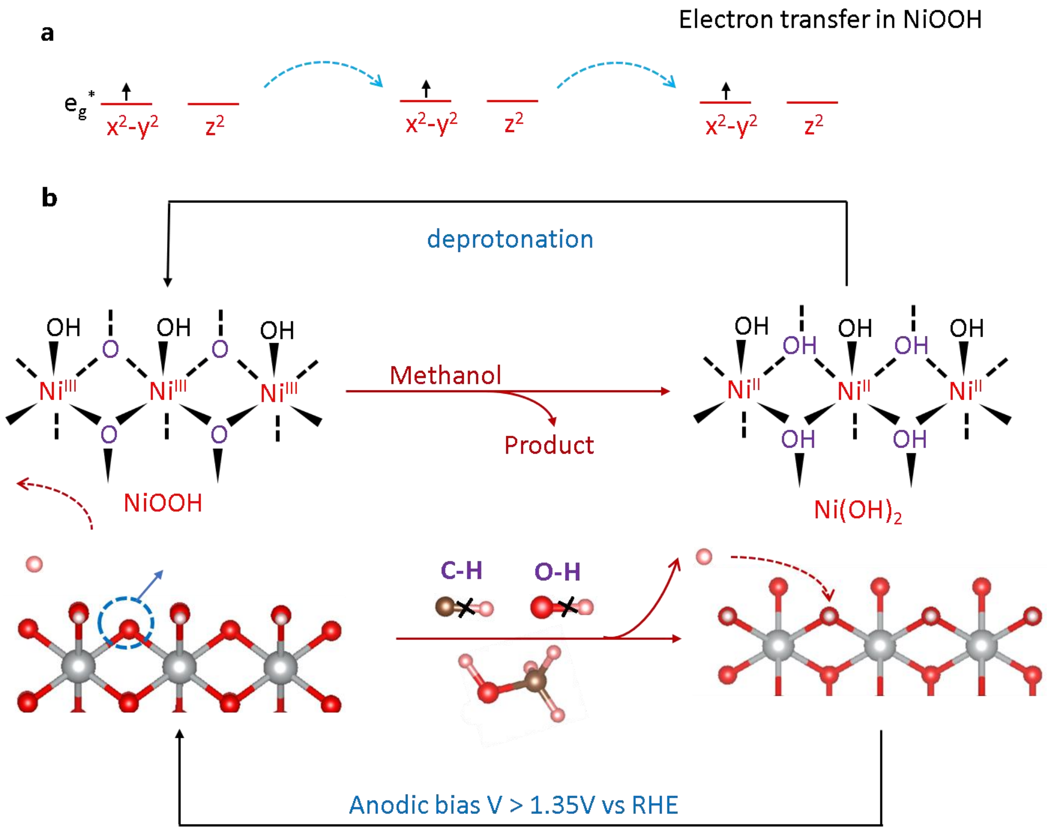


**Supplementary Figure 14.** (a) the electron transfer pathway in NiOOH; (b) the methanol oxidation process of traditional $Ni(OH)_2$. It indicated, there was only one spin up electron on NiOOH $e_g^*$ orbital. As the orbitals of $x^2$-$y^2$ and $z^2$ are nearly degenerate configuration (here we ignored the small effect of hydrogen), it would allow effective electron transfer into the adjacent $e_g^*$ orbital. At the same time, the methanol oxidation process of traditional $Ni(OH)_2$ could be summarized as: a) nickel oxidation, $Ni^{2+}$ to $Ni^{3+}$; b) methanol oxidation $Ni^{3+}$+ $CH_3OH$ → $Ni^{2+}$ + products.

## Supplementary Tables

| Anode | Cathode | Open-circuit voltage (V) |
|---|---|---|
| Ni metal | Pt/C | 0.002 |
| NiFe LDH | Pt/C | 0.016 |
| α-$Ni(OH)_2$ | Pt/C | 0.005 |
| β-$Ni(OH)_2$ | Pt/C | 0.007 |
| NR-$Ni(OH)_2$ | Pt/C | 0.580 |
| Pt/C | Pt/C | 0.650 |
| PtRu/C | Pt/C | 0.700 |

**Supplementary Table 1. The open circuit voltage (OCV) measurement of α/β-$Ni(OH)_2$, Ni metal, NiFe LDH catalysts, NR-$Ni(OH)_2$, standard Pt/C and PtRu/C catalysts.** The OCV measurement was evaluated by a homemade membrane-DMFC, which consists of two electrodes in the 1M KOH + 1M $CH_3OH$ electrolyte. The electrolyte is saturated with dissolved oxygen by continuously bubbling $O_2$ into the solution. The results indicated, nearly no electric output was seen for α/β-$Ni(OH)_2$, Ni metal and NiFe LDH catalysts, while high OCV values (OCV>0.55 V) were found in NR-$Ni(OH)_2$, Pt/C and PtRu/C catalysts.

**Supplementary Discussions**

**The detailed MOR activity calculations.**

Step 1 $CH_3OH$ adsorption. For methanol, the calculated bond lengths are 1.098 Å for C-H, 1.430 Å for C-O and 0.972 Å for O-H, agreeing well with reported experimental values of 1.09, 1.43 and 0.95 Å (*5*), receptively. The adsorption of methanol results from the lone pair electrons of oxygen bonding to a metallic surface or atoms, forming a weak adsorption state. Our calculation results show that methanol adsorption on NR-$Ni(OH)_2$ prefers the four-coordinated Ni at the edges. The $CH_3OH$ molecule is circled.

Step 2 $CH_3OH \rightarrow CH_3O^*$. The initial state is the adsorption of $CH_3OH$, and the final state is the adsorption of $CH_3O^*$, as shown below. The methoxy ($CH_3O$) (right) also prefers to be adsorbed at the Ni atom sites, with Ni-O bond lengths of 1.845 and 1.862 Å. H was adsorbed at the neighboring O atom. It should be noted that we also considered the possibility of $CH_3OH$-$CH_2OH^*$. However, the required energy for breaking the C-H bond is much higher than that for the O-H bond. Therefore, we did not consider the decomposition process of $CH_3OH$-$CH_2OH^*$ further.

Step 3 $CH_3O^* \rightarrow CH_2O^*$. The initial state is the adsorption of $CH_3O$, and the final state is the adsorption of $CH_2O^*$. $CH_3O^*$ is decomposed to $CH_2O^*$ by breaking a C-H bond, see below. The reaction process is calculated to be exothermic by 2.03 eV with an energy barrier of 0.91 eV. $CH_2O$ prefers to be adsorbed at a Ni atom site and the released H prefers to adsorb at the neighboring O atom site. The Ni-O bond length is slightly increased to 1.996 Å.

Step 4 $CH_2O^* \rightarrow CHO^*$. The initial state is now the adsorption of $CH_2O$, and the final state is the adsorption of $CHO^*$. $CH_2O^*$ is decomposed to $CHO^*$ by breaking another C-H bond. The reaction process is calculated to be endothermic by 0.22 eV with an energy barrier of 1.32 eV. CHO prefers to be adsorbed at a Ni atom site and H prefers to be adsorbed at the neighboring O atom site. The Ni-O bond length is slightly increased to 2.042 Å.

Step 5 $CHO^* \rightarrow CO^*$. The initial state is the adsorption of CHO and the final state is the adsorption of CO. CHO is decomposed to CO+H by breaking yet another C-H bond. The reaction process is calculated to be exothermic by 2.43 eV with an energy barrier of 0.27 eV. CHO also prefers to be adsorbed at a Ni atom site and H again prefers to be adsorbed at an O atom site. The O-Ni bond length is further increased to 2.210 Å. Based on our simulation results, this reaction process should be immediate. More interestingly, we found that the C atom bonds with Ni atoms instead of O atoms once H is decomposed from CHO.

Step 6 $CO^* \rightarrow COOH^*$. With C now bonding to Ni, the O lone pairs are exposed. In the alkaline reaction environment with abundant hydroxyl (-OH), hydroxyl adsorption at O sites will be rapid. The initial state is therefore the adsorption of CO+OH and the final state is the adsorption of COOH. The reaction process is calculated to be exothermic by 2.32 eV with an energy barrier of 0.25 eV. COOH prefers to adsorb at the Ni atom site and the C-Ni bond length is 1.910 Å. Based on our simulation results, this reaction process should arise immediately after step 5.

Step 7 $COOH^* \rightarrow CO_2^*$. The initial state is the adsorption of COOH and the final state is the adsorption of H and release of $CO_2$. In this process, COOH is decomposed to $CO_2$ and H becomes adsorbed at an O site, as yellow arrow indicated. The reaction process is calculated to be exothermic by 2.23 eV with an energy barrier of 0.21 eV. Based on our calculated results, it can be seen that this reaction would arise immediately. The C-O bond length in the adsorbed $CO_2$ is 1.174 Å, which is nearly the same as that in a $CO_2$ molecule. The optimized C-O bond length in $CO_2$ gas is 1.177 Å. Therefore, we believe that the final reaction product is $CO_2$ gas and adsorbed protons. The low kinetic energy and exothermic reaction imply this step would be completed rapidly.

**Analysis on hysteresis of CV curves**

I*n-situ* FTIR combining DFT (Figure 2) and electrochemical impedance spectroscopy (Supplementary Figure 8) were employed to study the hysteresis in the CV curves. In *in-situ* FTIR, when potential was above 0.6 V, the amount of $CH_3O^*$ dramatically increased (Figure 2c), suggesting its fast accumulation and thus the start of the reaction, which agrees well with the onset potential of the forward oxidation peak shown in Figure 1a. The accumulation of $CH_3O^*$ implies that the generation of $CH_3O^*$ is the rate determining step of the reaction. However, when the potential was increased to 1.0 V, a sudden decrease in $COOH^*$ consumption rate was observed (Figure 2d), indicating that the rater determining step was relevant to $COOH^*$ when the potential was above 1.0 V. The DFT calculations revealed that both the O-H and C-H chemical bonds required relatively higher energy barriers. Thus, it is believed that the rate determining step should be $COOH^*$ generation when potential above 1.0 V, which required $CH_3O^*$ to be further dehydrogenated (C-H bond breaking). Based on these, it is believed that the potential difference between forward and backward peaks is due to the change in the rate determining step from O-H chemical bonds breaking to C-H chemical bonds breaking during the dehydrogenation process. The change in rate-determining steps is also confirmed in the electrochemical impedance spectroscopy measurements (Supplementary Figure 8).

**Supplementary References**


1. Wu, D., Zhang, W. & Cheng, D. Facile synthesis of Cu/NiCu electrocatalysts integrating alloy, core-shell, and one-dimensional structures for efficient methanol oxidation reaction. *ACS Appl. Mater. Interfaces* **9,** 19843-19851 (2017).

2. Danaee, I., Jafarian, M., Mirzapoor, A., Global, F. & Mahjani, M. G. Electrooxidation of methanol on NiMn alloy modified graphite electrode. *Electrochimica Acta.* **55,** 2093-2100 (2010).

3. Abdel Rahim, M. A., Adbel Hameed, R. M. & Khalil, M. W. Nickel as a catalyst for the electro-oxidation of methanol in alkaline medium. *J. Power. Sources.* **134,** 160-169 (2004).

4. Chung, D. Y., Lee, K. & Sung, Y. Methanol electro-oxidation on the Pt surface: Revisting the cyclic voltammetry interpretation. *J. Phys. Chem. C* **120,** 9028-9035 (2016).

5. Mills, G., Jonsson, H. & Schenter, G. K. Reversible work transition state theory: application to dissociative adsorption of hydrogen, *Surf. Sci.* **324,** 305-337 (1995).
